# Health Tract, No. 24

**Published:** 1865

**Authors:** 


### HEALTH TRACT, No. 24.

### SOUR STOMACH.

NATURE provides a liquid (the gastric juice) in the stomach, sufficient to dissolve as much food as the system requires, and no more. Whatever is eaten beyond what is needed has no gastric juice to dissolve it, and being kept at the temperature of the stomach, which is about a hundred degrees, it begins to decompose—that is, to sour—in one, two, three, or more hours, just as new cider begins to sour in a few hours. In the process of souring, gas is generated as in the cider-barrel, the bung is thrown out, and some of the contents run over at the bung-hole, because in souring, the contents expand, and require more room. So with the stomach. It may be but partially filled by a meal; but if more has been swallowed than wise nature has provided gastric juice for, it begins to sour, to ferment, to distend, and the man feels uncomfortably full. He wants to belch. That gives some relief. But the fermentation going on, he gets the "belly ache" of childhood or some other discomfort, which lasts for several hours, when nature succeeds in getting rid of the surplus, and the machinery runs smoothly again. But if these things are frequently repeated, the machinery fails to rectify itself, looses the power of reädjustment, works with a clog, and the man is a miserable dyspeptic for the remainder of life; and all from his not having had wit enough to know when he had eaten a plenty, and being foolish enough, when he had felt the ill effects of thus eating too much, to repeat the process an indefinite number of times; and all for the trifling object of feeling good for the brief period of its passing down the throat. For each minute of that good he pays the penalty of a month of such suffering as only a dyspeptic can appreciate. When the dyspepsia is in the stomach, there is belching or eructations; when in the bowels, the gases pass downwards, until chronic diarrhœa sets in, with its relentless wasting away of flesh and strength, and finally of life itself; or fearful neuralgias rack the whole frame, first in one place, then in another, often making a curse of that life which ought to have been a blessing. When there is habitual acidity or wind after meals, it is the result of an error in eating; oftener in quantity than quality; and common-sense dictates taking less and less each meal, and adhering to the amount which can be taken without any subsequent discomfort whatever.

